# Distribution of *Lewis* and *Secretor* polymorphisms and corresponding CA19‐9 antigen expression in a Chinese population

**DOI:** 10.1002/2211-5463.12278

**Published:** 2017-10-04

**Authors:** Meng Guo, Guopei Luo, Renquan Lu, Weizhong Shi, He Cheng, Yu Lu, Kaizhou Jin, Chao Yang, Zhengshi Wang, Jiang Long, Jin Xu, Quanxing Ni, Chen Liu, Xianjun Yu

**Affiliations:** ^1^ Department of Pancreatic Surgery Fudan University Shanghai Cancer Center China; ^2^ Department of Oncology Shanghai Medical College Fudan University Shanghai China; ^3^ Pancreatic Cancer Institute Fudan University Shanghai China; ^4^ Department of Clinical Laboratory Fudan University Shanghai Cancer Center China

**Keywords:** Chinese, FUT2, FUT3, genotype, SNP

## Abstract

The *Lewis* (*FUT3*) and *Secretor* (*FUT2*) genes, corresponding to secretion of Lewis ABO (H) histo‐blood group antigen CA19‐9, are highly polymorphic with differences between populations. In this study, the *FUT3* and *FUT2* genes in 316 Chinese participants were sequenced to detect polymorphisms, and the associated CA19‐9 antigen secretion was also measured. In total, 14 genotypes of *FUT3* and 10 genotypes of *FUT2* were verified. *Le*/*Le*,* Le*/*le* ^59,508^ and *Le*/*le* ^59^ were the main genotypes of *FUT3* with frequencies of 53.2%, 10.7% and 3.5%, respectively. *Se*/*Se*,* Se*/*se* ^385^ and *se* ^385^/*se* ^385^ were the main genotypes of *FUT2*, with frequencies of 21.4%, 18.6% and 16.2%, respectively. The alleles *le* ^1067^ and *le* ^508^ were found extensively in the Chinese population, and the frequency of allele *se* ^385^ was shown to be higher than previously reported. Phenotype analysis revealed that 9.8% of individuals were the Lewis‐negative type and 22.5% were the secretor‐negative type. Combined phenotypes showed that 3.2% of participants were of ‘double‐negative’ phenotype (le, se) and 19.3% were of single dominant non‐secretor phenotype (Le, se). Serum Lewis antigen CA19‐9 levels were significantly different between subgroups and consistent with the defined phenotype. Our study revealed the unique distribution of *Lewis* and *Secretor* polymorphisms in a large Chinese population, and decoded the combined genotypes of the two CA19‐9‐related genes.

**Abbreviations**
*Le*: *Lewis* (*FUT2*) gene negative genotype
*Le*: *Lewis* (*FUT3*) gene positive genotype
*se*: *Secretor* (*FUT2*) gene negative genotype
*Se*: *Secretor* (*FUT2*) gene positive genotype
SNP: single nucleotide polymorphism

The synthesis of the Lewis ABO (H) histo‐blood group antigens requires multiple specific glycosyltransferases 1, 2, 3. The *FUT3* (*Lewis*) and *FUT2* (*Secretor*) genes encode an α‐(1,3/4)‐fucosyltransferase and an α‐(1,2)‐fucosyltransferase, respectively, which regulate fucose‐carbohydrate antigen synthesis by adding a fucose to precursor substrate 3, 4, 5, 6. Cooperation of the two fucosyltransferases ultimately regulates the expression of the histo‐blood group antigens, including CA19‐9 (sLe^a^), in body fluids and on the surface of epithelial cells. A single nucleotide polymorphism (SNP) of *FUT3* and *FUT2* is prevalent in multiple populations and dramatically determines the fucosyltransferase activities 4, 5, 6, 7, 8. Distribution of the *FUT3*/*FUT2* genotypes exhibits ethnic heterogeneity 9, 10 and is strongly associated with a wide range of human diseases 8, 11, 12, 13, 14.

The α‐(1,3/4)‐fucosyltransferase‐encoding gene *FUT3*, also known as the *Lewis* gene (*Le*), is essential for the synthesis of Lewis histo‐blood group antigens. The fucosyltransferase diverts a fucose to either the type 1 precursor or the H type 1, to form Le^a^ or Le^b^, respectively. Mutations in the *FUT3* gene may result in the Lewis‐null phenotype (le) 7, 15, 16, 17, 18. SNPs rs28362459 (T59G), rs812936 (T202C), rs778986 (C314T), rs3745635 (G508A) and rs3894326 (T1067G) of the *FUT3* gene are the most common polymorphic loci in Asians 7, 10, 15, 18, 19. Substitution of amino acids caused by mutations T202C, C314T, G508A and T1067G leads to inactivation of the FUT3 enzyme, and mutation T59G reduces the availability of α‐(1,3/4)‐fucosyltransferase 2, 6, 11.

The *FUT2* gene, also known as the *Secretor* gene (*Se*), determines the secretion status of histo‐blood group antigens 20. It encodes an α‐(1,2)‐fucosyltransferase (FUT2) that adds a fucose onto the type 1 precursor to form H type 1, the precursor of Le^b^. According to previous reports, non‐secretor phenotypes in Western populations are mainly caused by a homozygous loss‐of‐function mutation of *FUT2* (rs601338, G428A) 5, 14, 21. However, the frequency of mutation G428A in Asians is much lower 20, 22, 23. Approximately 20% of the Asian population are non‐secretors, and homozygous missense at site 385 (rs1047781, A385T), which is the primary mutation, results in the non‐secretor phenotype 20, 22, 23, 24. Additionally, a synonymous mutation (rs281377, T357C) and a non‐synonymous mutation (rs602662, G739A) have been shown to be common in Asians 12, 20, 23, 24. The fusion gene (*se* ^fus^) was found in Japanese and Korean populations, but was not detected in the Chinese population 9, 23.

Lewis‐negative individuals (the *le*/*le* genotype) have the Lewis (FUT3)‐negative phenotype, Le (a−b−), irrespective of the *Se* genotype. However, Lewis‐positive individuals (the *Le*/*Le* and *Le*/*le* genotypes) are divided into three Lewis‐secretor phenotypes according to distinct *Secretor* genotypes as follows: (a) Le (a−b+) secretors with the *Se*/*Se* or *Se*/*se* genotype; (b) Le (a+b−) non‐secretors with the *se*/*se* genotype; and (c) Le (a+b+) partial secretors having homozygosity for the weak *Secretor* allele 1, 2, 20, 25. Nucleotide substitutions inactivating the *FUT2*/*3* genes have been found within various populations, and the phenotypes have been determined by the ethnic group‐specific genotypes 1, 8, 16, 19, 24, 25. Both genotypes of *Lewis* and *Secretor* are crucial for an individual's phenotype formation, and the serum CA19‐9 value will directly reflect the differences among individuals.

Knowledge of the polymorphisms of the *Lewis* and *Secretor* genes in the Chinese population will help in classifying the subgroups and defining an accurate condition for normal phenotypes. In this study, we aimed to examine the prevalence of the five major nucleotide polymorphisms of the *FUT3* gene and the four main variation types of the *FUT2* gene in a Chinese population. Comparison of serum CA19‐9 expression between each phenotype was performed as well. Distribution of the Lewis‐negative and secretor‐negative phenotypes in the Han ethnic population was also evaluated and compared with previous results in other populations. Since they are based on a reliable method, we expect the results will provide an important reference for disease diagnosis and therapy in the Chinese population.

## Materials and methods

### Participants and genomic DNA isolation

Blood samples were obtained from 316 unrelated and randomly selected healthy individuals of Han ethnicity in the eastern region of China. Either their birthplace or the paternal origin of the participants in this study was in mainland China. Oral informed consent was obtained from all participants in this study. We collected the peripheral blood in tubes containing ethylene diamine tetraacetic acid and isolated the white blood cells. Genomic DNA was extracted from white blood cells using a QIAamp DNA Blood Mini kit (Qiagen, Inc., Hilden, Germany) according to the manufacturer's instructions.

### PCR amplification of *FUT3* and *FUT2* genes

Both *FUT3* and *FUT2* genes have no intron in the open reading frame and fewer reactions are capable of amplification of the complete mutation region. Three pairs of PCR primers respectively specific for the *FUT3* and *FUT2* gene segments are shown in Table S1, and sequence design partly referred to those previously reported 16, 26. For each segment amplification, 20 ng of genomic DNA was combined with the primers (7.5 μm for forward and reverse) in a PCR system of final volume 25 μL. Each PCR system contained 5 mm dNTPs, 37.5 mm MgSO_4_, 2.5 μL 10 × PCR buffer (particular for KOD ‐Plus‐ Neo) and 0.5 U KOD ‐Plus‐ Neo (Toyobo Co., Osaka, Japan). Thirty cycles were run (2 min at 94 °C, 10 s at 98 °C, 30 s at *T*_m_ and 30 s at 68 °C, where *T*_m_ for 385F/385R is 62 °C, for 508F/1067R is 65.5 °C and for 21F/21R is 60 °C), and the products were isolated from agarose gels for sequencing.

### Direct DNA sequencing and genotyping

The purified amplification products were sequenced directly for *FUT3* and *FUT2* genotyping. The anterior half‐segment of *FUT3* concluding at the 59, 202 and 314 position was directly sequenced with primers 385F and 385R. The bottom half‐segment of *FUT3* concluding at the 59, 202 and 314 position was directly sequenced with primers 508F and 1067R. The complete segment of *FUT2* concluding at the 357, 385, 428 and 739 position was directly sequenced with primers 508F and P1R. The dideoxynucleotide termination sequencing reaction was performed by using the ABI BigDye Terminator cycle sequencing system, and the DNA sequence was analyzed by an ABI PRISM 3730 instruments (Applied Biosystems, Carlsbad, CA, USA). Sequencing data were generated by the ABI 3730 Genetic Analyzer platform and were analyzed using chromas software 27. The genotype present at each SNP site was directly determined by one or two different color peaks on the electropherogram.

### CA19‐9 antigen measurement and statistical analysis

Serum CA19‐9 antigens were measured using an electrochemiluminescence immunoassay on the Roche Cobas e601 (Roche MODU D + P model, D2400‐P800) immunoassay analyzer (Roche Diagnostics, Mannheim, Germany). An obtained CA19‐9 value of less than 0.06 U·mL^−1^ was considered to be undetectable. The frequencies of the gene polymorphisms, as well as the genotypes and phenotypes, were determined by description analysis. Difference of CA19‐9 values among each subgroup were tested by one‐way analysis of variance and Student's *t* test was used to compare between each single group. Statistical analyses were performed with spss Statistics 19 (IBM Corp., Armonk, NY, USA) and *P* < 0.05 was defined as statistically significant.

### Ethical approval

All procedures performed in studies involving human participants were in accordance with the ethical standards of the Fudan University Shanghai Cancer Center.

## Results

### Distribution of *FUT3* and *FUT2* gene polymorphisms in a Chinese population

We amplified the coding regions containing the SNPs in the *FUT3* (*Lewis*) and *FUT2* (*Secretor*) genes and examined the respective polymorphisms. For the *Lewis* gene, nucleotide 59T>G was the most prevalent mutation, with a frequency of 39.87% (7.59% homozygous and 32.28% heterozygous). Variations 508G>A (25.00%) and 1067T>A (8.23%) manifested in a homozygous form. Variations 202T>C and 314C>T had complete linkage in 18 individuals (5.70%) and no homozygous alleles were encountered (Table 1). For the *Secretor* gene, four prevalent polymorphisms were also investigated. It is known that the most frequent *Secretor* gene mutations are detected in Asians, which as a homozygous mutation causes a non‐secretor phenotype 20, 23, 24. 385A>T was detected in 222 individuals (70.25%) with a *T* allele frequency of 46.04%. Variation 357A>T was detected with a frequency of 24.68%, and induced a synonymous mutation in *Secretor*. Mutation at nucleotides 428 and 739 were rarely detected (1.27%) and showed complete linkage (Table 1).

**Table 1:** Distribution of SNPs and the allele frequencies

Nucleotide position		SNP			Allele	
		Wild‐type	Heterozygous mutant	Homozygous mutant	Primary allele	Variant allele
		*n* (%)	*n* (%)	*n* (%)	*n* (%)	*n* (%)
*Lewis*	59	TT	TG	GG	*T*	*G*
		190 (60.13)	102 (32.28)	24 (7.59)	482 (76.27)	150 (23.73)
	202	TT	TC	CC	*T*	*C*
		298 (94.30)	18 (5.70)	0 (–)	614 (97.15)	18 (2.85)
	314	CC	TC	TT	*C*	*T*
		298 (94.30)	18 (5.70)	0 (–)	614 (97.15)	18 (2.85)
	508	GG	GA	AA	*G*	*A*
		237 (75.00)	64 (20.25)	15 (4.75)	538 (85.13)	94 (14.87)
	1067	TT	TA	AA	*T*	*A*
		290 (91.77)	23 (7.28)	3 (0.95)	603 (95.41)	29 (4.59)
*Secretor*	375	TT	TC	CC	*T*	*C*
		238 (75.32)	70 (22.15%)	8 (2.53)	546 (86.39)	86 (13.61)
	385	AA	TA	TT	*A*	*T*
		94 (29.75)	153 (48.42%)	69 (21.84)	341 (53.96)	291 (46.04)
	428	GG	GA	AA	*G*	*A*
		312 (98.73)	4 (1.27%)	0 (–)	628 (99.37)	4 (0.63)
	739	GG	GA	AA	*G*	*A*
		312 (98.73)	4 (1.27%)	0 (–)	628 (99.37)	4 (0.63)

### Haplotype analysis of *FUT2* and *FUT3* genes

Based on combinations of known alleles and their secretor phenotypes, seven haplotypes of the *FUT3* gene and five haplotypes of the *FUT2* gene were estimated according to each allelic polymorphism. *Le* and *le* ^59,508^ were present as the most common haplotypes in *FUT3*, with a frequency of 72.94% and 14.72%, respectively. Haplotypes *le* ^59^ and *le* ^59,1067^ appeared with an equal frequency of 4.75%. Isolated *le* ^508^ and *le* ^1067^ alleles were rare (0.32% and 0.16%, respectively) and *le* ^59,508,1067^ was not detected. *le* ^202,314^ combining the 202T>C and 314C>T variations was only found as a co‐occurrence, with a frequency of 2.85%. (Table 2).

**Table 2:** Distribution of *Lewis* and *Secretor* genotypes

*Lewis* (*FUT3*)					*Secretor* (*FUT2*)				
Genotype	Phenotype	Distribution *n* (%)	Allele	Frequency (%)	Genotype	Phenotype	Distribution *n* (%)	Allele	Frequency (%)
*Le*/*Le*	Le	176 (55.70)	*Le*	72.94	*Se*/*se* ^385^	Se	114 (36.08)	*Se*	46.20
*Le*/*le* ^59,508^	Le	56 (17.72)	*le* ^59,508^	14.72	*se* ^385^/*se* ^385^	se	69 (21.84)	*se* ^385^	40.19
*Le*/*le* ^59^	Le	24 (7.59)	*le* ^59^	4.75	*Se*/*Se*	Se	55 (17.41)	*se* ^357,385^	5.85
*le* ^59,508^/*le* ^59,508^	le	15 (4.75)	*le* ^59,1067^	4.27	*Se*/*se* ^357,385^	Se	36 (11.39)	*Se* ^357^	7.12
*Le*/*le* ^59,1067^	Le	15 (4.75)	*le* ^202,314^	2.85	*Se*/*Se* ^357^	Se	31 (9.81)	*se* ^357,428,739^	0.63
*Le*/*le* ^202,314^	Le	11 (3.48)	*le* ^1067^	0.32	*Se* ^357^/*Se* ^357^	Se	6 (1.90)		
*le* ^59,1067^/*le* ^59,508^	le	5 (1.58)	*le* ^508^	0.16	*se* ^385^/*se* ^357,428,739^	se	2 (0.63)		
*le* ^59^/*le* ^202,314^	le	4 (1.27)			*Se* ^357^/*se* ^357,385^	Se	1 (0.32)		
*le* ^59,1067^/*le* ^59,1067^	le	3 (0.95)			*Se* ^357^/*se* ^357,428,739^	Se	1 (0.32)		
*le* ^59,508^/*le* ^202,314^*	le	2 (0.63)			*Se*/*se* ^357,428,739^	Se	1 (0.32)		
*Le*/*le* ^1067^	Le	2 (0.63)							
*le* ^59^/*le* ^59^	le	1 (0.32)							
*le* ^59,1067^/*le* ^202,314^*	le	1 (0.32)							
*Le*/*le* ^508^	Le	1 (0.32)							

Presumed genotypes.

For the *FUT2* gene, *Se* and *se* ^385^ were the most prevalent haplotypes at a frequency of 46.20% and 40.19%, respectively, and thus accounted for 86% of all allele counts. The rarely detected *Se* ^357^ and *se* ^357,385^ were present at a frequency of 7.12% and 5.85%, respectively. We also noticed that sole variations of *se* ^428^ and *se* ^739^ were hardly encountered in the Chinese population and only appeared as allele *se* ^357,428,739^ at a low frequency of 0.63% (Table 2).

### Distribution of *FUT2* and *FUT3* genotypes

**Figure 1:**
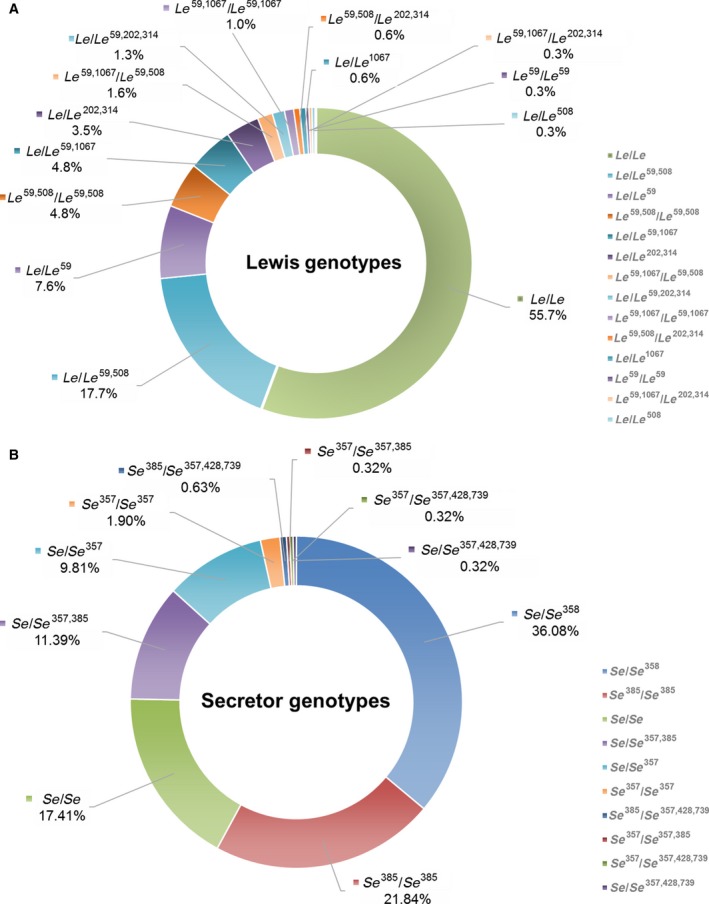
Distribution and frequencies of the *FUT3* (*Lewis*) and *FUT2* (*Secretor*) genotypes in a Chinese population. Doughnut charts representing genotypes for (A) *FUT3* (*Lewis*) and (B) *FUT2* (*Secretor*).

For *FUT3* (*Lewis*) genotypes, *Le*/*Le*,* Le*/*le* ^59,508^ and *Le*/*le* ^59^ were demonstrated to be the most common positive genotypes (giving a Lewis‐functional phenotype), and were detected with a frequency of 55.70%, 17.72% and 7.59%, respectively. Thirty‐one individuals (9.82%) exhibited Lewis‐negative genotypes (giving the Lewis‐null phenotype), in which *le* ^59,508^/*le* ^59,508^ (2.17%) was shown to be the most prevalent (Fig. 1A and Table 2).

For *FUT2* (*Secretor*) genotypes, allele *se* ^385^ was the major factor giving rise to 97% of non‐secretor phenotypes. In the secretor‐positive cohort, *Se*/*se* ^385^ was confirmed to be the most prevalent genotype, with a frequency of 36.08%, and individuals with the *Secretor* wild‐type (*Se*/*Se*) were at 17.41%. The non‐secretor phenotype was found at a frequency of 22.46% and mostly (21.84%) was identified as *se* ^385^/*se* ^385^. Another non‐secretor genotype, *se* ^*385*^ */se* ^*357*,*428*,*739*^, was much rarer (0.63%). *se* ^428^ was previously reported as the major mutation to cause the non‐secretor phenotype and to be widely distributed in other races 28, but was not detected in Chinese. In addition, variation at position 428 was also present in conjugation with the 357 and 739 variation, and completely co‐present with 739 (Fig. 1B and Table 2).

A cluster analysis based on each SNP was performed by combining genotypes of the studied objects (Fig. 1). In total, 55 combined genotypes were encountered of which 18 types (84.9%) were present with a frequency greater than 1% (Table 3). As shown in the genotypes distributions in Fig. 1, subjects homozygous for the functional allele are marked as *Le*/*Le* or *Se*/*Se*, homozygous mutated loss‐of‐function alleles are denoted as *le*/*le* or *se*/*se*, and heterozygotes mutated genotypes are represented by *Le*/*le* or *Se*/*se*. The particular nucleic acid site variations of the *Lewis*,* Secretor* and combined genotypes is showed in Table S2.

**Table 3:** Distribution of combined genotype of the *FUT3* and *FUT2* genes and the corresponding phenotype. *Le*,* Se* represents the double‐positive genotype; *Le*,* se* represents Lewis positive and synchronously secretor negative; *le*,* Se* represents Lewis negative and synchronously secretor positive; *le*,* se* represents the double‐negative genotype. *Genotypes with frequency less than 1% were omitted

Combined *Lewis* and *Secretor*	Phenotype	Distribution* *n* (%)	Combination	
			Phenotype	Frequency (%)
*Le*/*Le* and *Se*/*se* ^385^	Le, Se	60 (19)	Le, Se	70.9
*Le*/*Le* and *se* ^385^/*se* ^385^	Le, se	38 (12)	Le, se	19.3
*Le*/*Le* and *Se*/*Se*	Le, Se	27 (8.5)	le, Se	6.6
*Le*/*le* ^59,508^ and *Se*/*se* ^385^	Le, Se	23 (7.3)	le, se	3.2
*Le*/*Le* and *Se*/*se* ^357,385^	Le, Se	23 (7.3)	Sum	100.0
*Le*/*Le* and *Se*/*Se* ^357^	Le, Se	20 (6.3)	**Particular**	
*Le*/*le* ^59^ and *Se*/*se* ^385^	Le, Se	13 (4.1)	**Phenotype**	**Frequency (%)**
*Le*/*le* ^59,508^ and *Se*/*Se*	Le, Se	11 (3.5)	Lewis‐null (*le*)	9.8
*Le*/*le* ^59,508^ and *se* ^385^/*se* ^385^	Le, se	9 (2.9)	Non‐secretor (*se*)	22.5
*le* ^59,508^/*le* ^59,508^ and *se* ^385^/*se* ^385^	le, se	7 (2.2)		
*le* ^59,508^/*le* ^59,508^ and *Se*/*se* ^385^	le, Se	5 (1.6)		
*Le*/*le* ^59,508^ and *Se*/*Se* ^357^	Le, Se	5 (1.6)		
*Le*/*le* ^59,508^ and *Se*/*se* ^357,385^	Le, Se	5 (1.6)		
*Le*/*le* ^59,508^ and *se* ^385^/*se* ^385^	Le, se	5 (1.6)		
*Le*/*le* ^59^ and *Se*/*Se*	Le, Se	5 (1.6)		
*Le*/*le* ^59,1067^ and *Se*/*se* ^385^	Le, Se	4 (1.3)		
*Le*/*le* ^202,314^ and *Se*/*Se*	Le, Se	4 (1.3)		
*Le*/*Le* and *Se* ^357^/*Se* ^357^	Le, Se	4 (1.3)		

### Combination of FUT2 and FUT3 phenotype and corresponding CA19‐9 value

**Figure 2:**
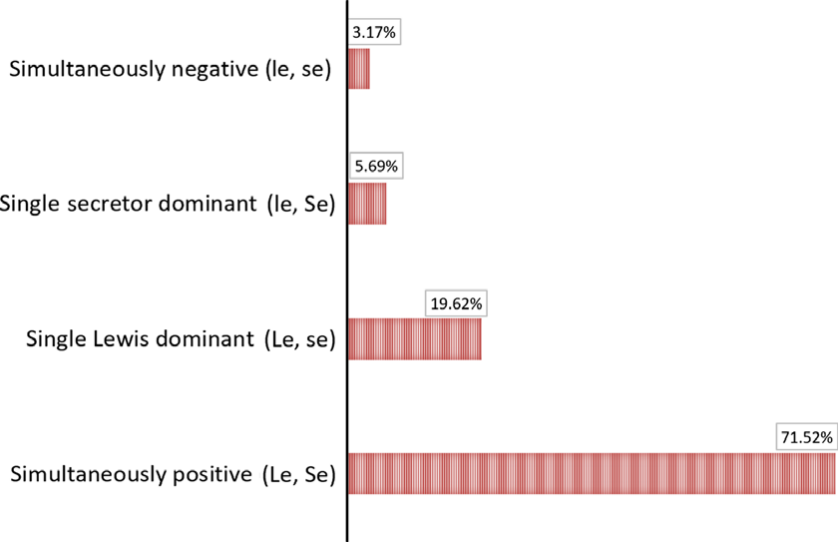
Distribution of combined phenotypes in Chinese population.

Corresponding to each genotype, the phenotype of single individuals was investigated. Four combined phenotypes were identified according to *Lewis* (*FUT3*) and *Secretor* (*FUT2*) genotypes (Fig. 2), namely Le, Se; Le, se; le, Se; and le, se. Among the population, 226 participants (71.52%) were detected as Le, Se (the double‐positive phenotype), which manifested as the highest frequency. Le, se (Lewis positive and synchronously secretor negative) and le, Se (Lewis negative and synchronously secretor positive) were present in 62 individuals at a frequency of 19.62% and in 18 individuals at a frequency of 7.28%, respectively. The double‐negative phenotype le, se was shown in 3.17% (10 of 316) of individuals. Table 3 summarized the 18 high‐frequency genotypes and corresponding phenotypes.

**Figure 3:**
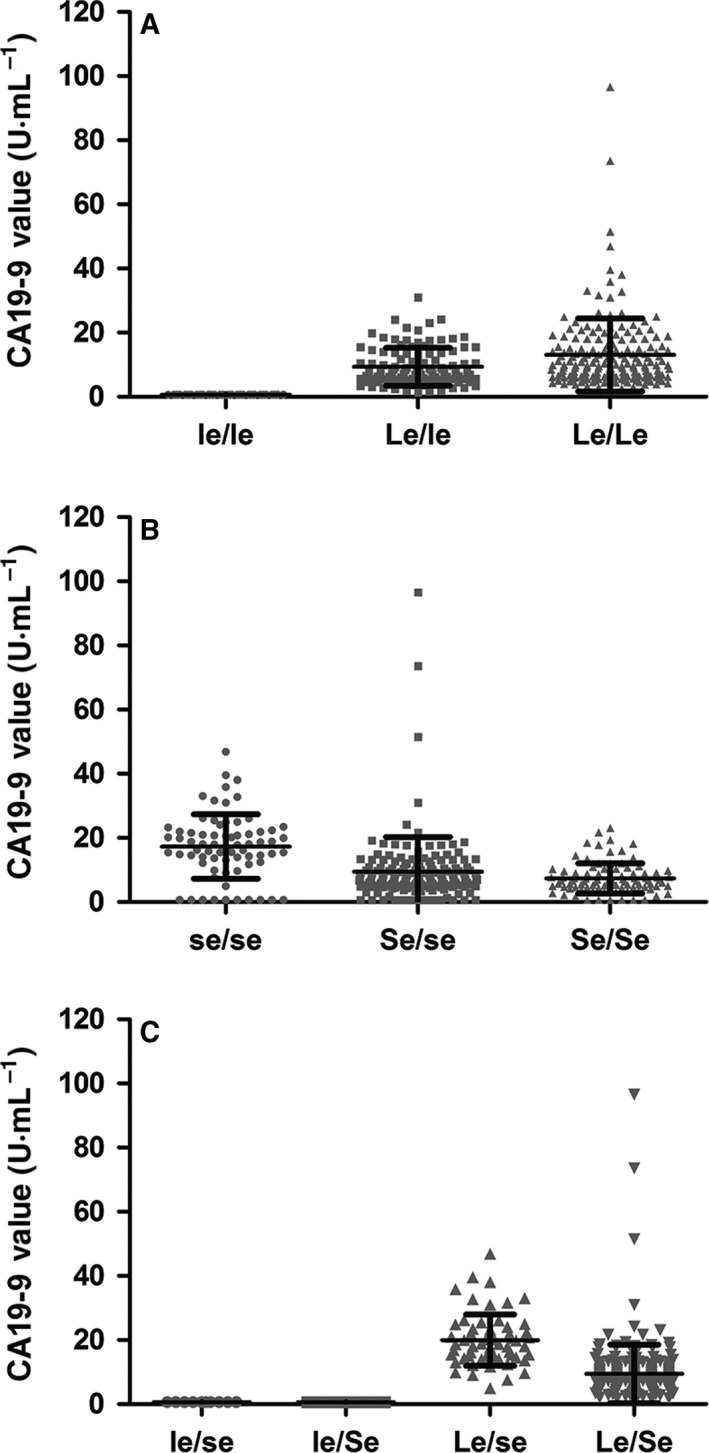
CA19‐9 values for different phenotypes. The comparison of serum antigen expression was conducted between Lewis phenotypes (A), secretor phenotypes (B) and combined phenotypes (C).

The serum CA19‐9 value of each participant was measured and compared between genotypes. Comparison between the Lewis‐functional phenotypes showed that CA19‐9 values in participants with the Le/Le genotype were significantly higher than ones with the Le/le genotype (mean 13.07 *vs* 9.37 U·mL^−1^, *P* < 0.05). Otherwise, CA19‐9 values in the Lewis‐null phenotype group were completely undetectable (CA19‐9 value < 0.06 U·mL^−1^) (Fig. 3A). Grouping by secretor phenotype, participants with the se/se phenotype showed a much higher CA19‐9 value (mean 17.27 U·mL^−1^) than the Se/se type (mean 9.43 U·mL^−1^) and Se/Se type (mean 7.38 U·mL^−1^) (*P* < 0.0001) (Fig. 3B). For the combined Lewis and secretor phenotypes, the Le/se group showed the highest CA19‐9 value compared with other groups (mean 20 U·mL^−1^, 95% CI: 17.95–22.05 U·mL^−1^) (*P* < 0.0001) (Table 4). Both le/se and le/Se types showed an undetectable CA19‐9 value (Fig. 3C and Table 4).

**Table 4:** Distribution of phenotypes and corresponding CA19‐9 values

Phenotype		No. of values	CA19‐9 value (U·mL^−1^)				95% CI	One‐way analysis of variance (*P* value)
			Min	Median	Max	Mean		
Lewis	le/le	31	< 0.6	< 0.6	< 0.6	< 0.6	< 0.6	< 0.0001
	Le/le	107	1.39	7.61	30.96	9.373	8.25–10.5	
	Le/Le	178	1.72	9.8	96.53	13.07	11.38–14.76	
Secretor	se/se	71	0.6	17.8	46.82	17.27	14.89–19.64	< 0.0001
	Se/se	153	0.6	7.38	96.53	9.43	7.71–11.15	
	Se/Se	92	0.6	6.495	23.03	7.383	6.41–8.359	
Combined	le/se	10	< 0.6	< 0.6	< 0.6	< 0.6	< .6	< 0.0001
	le/Se	22	< 0.6	< 0.6	< 0.6	< 0.6	< 0.6	
	Le/se	61	4.94	18.72	46.82	20	17.95–22.05	
	Le/Se	224	1.39	7.375	96.53	9.417	8.22–10.62	

### Population differentiation of the genotype in *FUT2* and *FUT3* alleles

**Figure 4:**
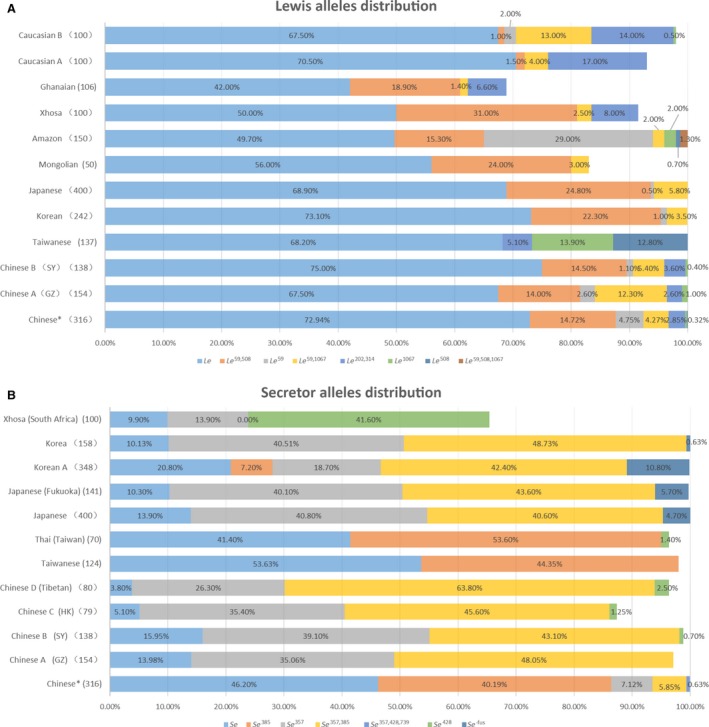
Distribution of genotypes and phenotypes in different ethnic and geographic groups. Comparison of *Lewis* (A) and *Secretor* (B) allele frequencies among different populations. (*Population in present study; a: Liu *et al*. 10; b: Liu *et al*. 19; c: Park *et al*. 29; d: Narimatsu *et al*. 25; e: Soejima *et al*. 31; f: Corvelo *et al*. 16; g: Pang *et al*. 15; h: Liu *et al*. 23; i: Yip *et al*. 22; j: Pang *et al*. 30 ; k: Chang *et al*. 26; l: Park *et al*. 9; m: Koda *et al*. 6; n: Liu *et al*. 28.)

In the present study, we encountered seven kinds of negative *Lewis* haplotypes and four kinds of negative *Secretor* haplotypes. Figure 4 presents the ethnic specificity of putative allelic frequencies among various populations including Korean 9, 29, Chinese 10, 22, 23, 30, Japanese 6, 25, Thai 26, Caucasian 15, 31 and others. Distribution of the *FUT3* genotype is approximatively consistent with reported data in Chinese and other Asian populations. Nevertheless, no Asian population study has confirmed the existence of *le* ^508^ previously, which is at a frequency of 0.16% in the present study and 0.70% in an Amazonian population 16. *le* ^59,508^ is abundantly distributed in most populations with a frequency from 14% to 31%, but rarely found in Caucasians (1.0% and 1.5%). In contrast, *le* ^202,314^ is present in Asians at a much low frequency compared with Caucasians 15, 31 (Fig. 4A).

The three most common *FUT2* alleles in the present study were *Se*,* se* ^385^ and *Se* ^357^, with a frequency of 46.20%, 40.19% and 7.12%, respectively, and the distribution is highly distinctive in multiple Asian populations including four Chinese groups 22, 23, 30, except for one group in Taiwan 26 (Fig. 4B). We have noticed that allele *se* ^385^ was found at an especially common rate in the current study compared with other populations. On the other hand, the frequency of *se* ^357,428^ in the present population is much lower, while the frequency of *Se* is much higher. No *se* ^fus^ was detected in Asians except for Japanese (4.7–5.7%). In contrast to Xhosa and Caucasians, *se* ^428^ in Asians is rare and previous studies revealed a very low frequency. However, our data showed that 428 absolutely conjugated with other mutations in Chinese rather than being an isolated allele. *se* ^357,428,739^ was not reported before in other studies, mainly accounted for by a deficiency in nucleotide 739 detection. The variations of *Lewis* and *Secretor* allele distributions among different populations are shown Table S3.

## Discussion

The distribution of genotype has been reported to have ethnic specificity, and genetic heterogeneity in Lewis‐null and non‐secretor individuals is obviously present in different populations 2, 15, 17, 18, 32. Even in the Chinese population, the frequency of phenotypes varies with subject selection, study range, detected position and investigation method 10, 19, 22, 26, 30. Integral and systemic comprehension of the *FUT3* (*Lewis*) and *FUT2* (*Secretor*) genotypes in the Chinese population has been insufficient.

In a large cohort of 316 Chinese participants, we performed accurate genotyping and a combined analysis of nine SNPs reported previously in the fucosyltransferase genes 8, 10, 16, 19, 23, 24. False‐negative reactions are often produced in Lewis blood typing due to weak hemagglutination reactions, which in the main is accounted for by adsorption of glycolipids and by erythrocyte levels 2, 16, 25. For further study, *FUT2*/*FUT3* genotyping is the most accurate method for distinguishing the phenotypes 6, 7, 11, 20. Accordingly, by direct sequencing, five major SNPs of the *FUT3* gene and four major SNPs of the *FUT2* gene were detected. Additionally, we also discovered 18 primary genotypes and four phenotypes in the Chinese cohort by cluster analysis. It was known that Lewis genotypes in Asians are inconsistent with the polymorphism in Europe and America, and our study has shown the unique distribution of the *FUT3*/*FUT2* gene polymorphism and allied genotype frequencies in a randomly recruited Chinese population. We analyzed the coding DNA sequence range and in *FUT3* did not detect any SNPs other than those previously reported, but three individuals with heterozygous 571C>T (rs1800028) in *FUT2* were detected.

In the present population, the ‘wild‐type’ (*Le*/*Le*) *FUT3* gene was detected at a frequency of 55.70%, which is consistent with previous reports in Chinese and Asians. Compared with Caucasians, allele *le* ^59,508^ is more abundant and *le* ^202,314^ is much rarer in Asians 15, 31. The Amazonian population shows a particular enrichment of the *le* ^59^ allele compared with other populations 16. Allele *le* ^1067^ and allele *le* ^508^ were reported to be absent in Asians; nevertheless, they were detected at the frequency of 0.32% and 0.16%, respectively, in the present Chinese population. This discrepancy could be attributed to the sensitivity of the detection method. The two alleles were reported with much higher frequency in a study that included three Asian populations, which was mostly caused by omitted the detection of *le* ^59^ 19.

The mutated allele *le* ^59^ was shown to have the highest prevalence (40.19%) in the present participants, which homozygotes generating an amino acid variation in the transmembrane domain. Variation 59T>G leading to an L20R amino acid substitution was reported to be responsible for a Lewis‐negative phenotype probably accounted for by a reduction in Golgi retention 3, 7, 33. We classified the isolated 59 mutated allele in the Lewis‐negative group (marked as *le* ^59^), even though multiple studies have counted the allele in the Lewis‐positive genotype 10, 15, 16. Variations of nucleotides 202 and 314 have usually been reported as present together 32, 34, and we found that both T202C and C314T were present at frequencies of 5.70% in the Chinese population and no isolated alleles were found. Similar correlation of two single mutations in *FUT2* was also demonstrated for 428 and 739, at a lower frequency of 1.27%.

Earlier reports have revealed that differential histo‐blood group antigen expression (such as CA19‐9) has been insufficiently attributed to *FUT3* variation. Enzyme FUT2 competitively binds the substrate from FUT3 and the genotype also influences histo‐blood group antigens status. Multiple polymorphic nucleotides in the *FUT2* gene reflecting the secretor (*Se*) and non‐secretor (*se*) type have been reported with ethnic specificity 5, 22. Homozygous 428G>A (non‐secretor allele *se* ^428^) was the first identified missense mutation and was reported in approximately 20% of Caucasians 5, 20. In Asians, variations 357C>T and 385A>T were common, but variation 428G>A was rare 9, 23, 24, 30. *se* ^385^ is reported as the most prevalent allele in Asian populations and showed the highest frequency (40.19%) in the current population. Although the allele *se* ^385^ was definitively associated with the non‐secretor phenotype, it was rarely present in European populations (at a frequency of 0.4%) 24. In comparison with other populations including Chinese, allele *Se* (wild‐type) and allele *se* ^385^ (non‐secretor type) appeared at obviously higher frequencies in the current study. Simultaneously, functional allele *Se* ^357^ and non‐secretor *se* ^357,385^ were much rarer than in other populations (Fig. 4B).

As the synthesized product, the Lewis ABO (H) histo‐blood group antigen is routinely utilized as a clinical diagnosis biomarker, especially for malignant gastrointestinal tumor indication 35. It was previously known that individuals with a Lewis‐negative blood group are not able to synthesize Lewis antigens, and that the *FUT2*/*3* gene status determines Lewis antigen synthesis and secretion 25, 36. In the present study, 9.8% of individuals detected as the *Lewis*‐negative genotype were confirmed as Lewis‐null and had undetectable serum sLe^a^ (CA19‐9) antigen, unrelated to the *Secretor* genotype. Moreover, we perceived no Le^a^, Le^b^ antigen synthase, and 22.5% of individuals detected as the *Secretor*‐negative genotype were perceived as non‐secretor and had no Le^b^ antigen synthase 25. Additionally, 19.3% of participants with the ‘single *Lewis* dominant genotype’ (*Le*,* se*) were detected with significantly more elevated sLe^a^ than those of the ‘simultaneously positive type’ (*Le*,* Se*) (mean 20 *vs* 9.42 U·mL^−1^, *P* < 0.0001). A classic study showed that nine groups divided from 400 normal individuals by *Le*/*Se* genotype were detected with discrepant serum CA19‐9 and DU‐PAN‐2 values, and DU‐PAN‐2 measurement was more useable for colorectal cancer diagnosis in *Le*‐negative patients 25. Recently, Wannhoff *et al*. 1 have found that differentiation between three FUT2/3 phenotypes improves the clinical practicability of gene‐based cut‐off values of CA19‐9 for cholangiocarcinoma diagnosis. The application of *FUT2*/*3* genotype‐based cut‐offs improved sensitivity to 82.4% and 100.0% in the intermediate and high biosynthesis groups, respectively 1. The indicator sensitivity of Lewis antigens would be significantly increased based on the determination of the *FUT2*/*3* genotype, which may have wide application in tumor diagnosis.

In conclusion, this genotyping study of *FUT2* and *FUT3* indicated the particular distribution of polymorphisms in the Chinese population. Clarifying individuals’ *FUT2*/*3* genotype by sequencing might facilitate clinical classification and accurate diagnosis. Frequencies of each allele and the association with phenotype should be investigated in an extended population and at additional polymorphism positions, and the characteristics of separate subgroups warrants further study.

## Author contributions

MG and CL designed the study; HC, CY and YL carried out experiments; RL and WS provided the materials; KJ, ZW and JL analyzed the sequencing data; QN and GL analyzed experimental results and conducted statistical analysis; MG and GL wrote the manuscript.

## Supporting information


**Table S1.** Primers sequence for PCR amplification of the *FUT3* (*Lewis*) and *FUT2* (*Secretor*) genes.
**Table S2.** Particular nucleic acid sites variation of *Lewis*,* Secretor* and combined genotypes.
**Table S3.** Comparison of *Lewis* and *Secretor* allele frequencies among different populations.
