# Supplementary material for: Distribution of Lewis and Secretor polymorphisms and corresponding CA19‐9 antigen expression in a Chinese population
Source: FEBS Open Bio. 2017 Oct 4;7(11):1660–71. doi: 10.1002/2211-5463.12278 (PMC5666394; doi:10.1002/2211-5463.12278)
Supplement: Supplementary file 1 — Table S1. Primers sequence for PCR amplification of the FUT3 (Lewis) and FUT2 (Secretor) genes. Table S2. Particular nucleic acid sites variation of Lewis, Secretor and combined genotypes. Table S3. Comparison of Lewis and Secretor allele frequencies among different populations. [file FEB4-7-1660-s001.doc]

**Title: Unique distribution of *Lewis* and *Secretor*** **polymorphisms in Chinese and the corresponding CA19-9 antigen expression**

Meng Guo^1,2,3¶^, Guopei Luo^1,2,3¶^, Renquan Lu^4^, Weizhong Shi^4^, He Cheng^1,2,3^, Yu Lu^1,2,3^, Kaizhou Jin^1,2,3^, Chao Yang^1,2,3^,Zhengshi Wang^1,2,3^, Jiang Long^1,2,3^, Jin Xu^1,2,3^, Quanxing Ni^1,2,3^, Chen Liu^1,2,3*^, Xianjun Yu^1,2,3*^

**Authors' Affiliations:**

^1^Department of Pancreas Surgery, Fudan University Shanghai Cancer Center; ^2^Department of Oncology, Shanghai Medical College, Fudan University;

^3^Pancreatic Cancer Institute, Fudan University;

^4^Department of Laboratory, Fudan University Shanghai Cancer Center, Shanghai 200032, China.

^*^Correspondence to: Xianjun Yu, MD, PhD, email: [yuxianjun@fudanpci.org](mailto:yuxianjun@fudanpci.org); Chen Liu, PhD, email: [liuchen@fudanpci.org](mailto:liuchen@fudanpci.org), No. 270, Dong’An Road, Xuhui District, Shanghai, 200032, China, Tel: 86-21-64175590-1308; Fax: 86-21-64031446;

^¶^Contributed equally for this work

**Running title:** Distribution of Lewis and Secretor SNPs in Chinese

**Keywords:** FUT2, FUT3, Genotype, SNP, Chinese

Supplementary Table 1. Primers sequence for PCR amplification of the FUT3 (Lewis) and FUT2 (Secretor) genes.

| **Gene** | **Mutation** | **Primer** | **Primer Sequence (5’ to 3’)** | **Position of Primer** |
| --- | --- | --- | --- | --- |
| **FUT3** | **T59G, T202C, C314T** | 385F | GGGTGCAGCCAAGCCACAA | 12bp-30bp of FUT3 |
|  |  | 385R | AGGTGGGAGGCGTGACTTAGG | 369bp-349bp of FUT3 |
|  | **G508A, T1067A** | 508F | ACTTGGAGCCACCCCCTAACTGCCA | 407bp-431bp of FUT3 |
|  |  | P1R | CGGCCTCTCAGGTGAACCAAGCCGCT | 7bp 431bp of FUT3FUT330000000000000000000000000000000000000000000000000000000000000000000000000000000000000000000000000000000000000(3’UTR)-1068bp of FUT3 |
| **FUT2** | **T357C, A385T, G428A** | 21F | CTAACGTGTCCCGTTTTCCTC | (-32bp)-(-12bp) of FUT2 (5’UTR) |
|  |  | 22R | CCACTCCGGCAGGAAGGC | 990bp-973bp of FUT2 |

Supplementary Table 2. Particular nucleic acid sites variation of Lewis, secretor and combined genotype.

| **FUT3**  **genotypes** | **SNPs status* in FUT3** | **FUT2** | **SNPs status* in FUT2** | **Combined genotypes** | **SNPs status* in the genes combination** |
| --- | --- | --- | --- | --- | --- |
| **Genotype** |  | **Genotype** |  |  |  |
| Le/Le | **59, 202, 314, 508, 1067** | Se/*se*^385^ | **357, 385T, 428, 739** | Le/Le&Se/*se*^385^ | **59, 202, 314, 508, 1067/ 357, 385T, 428, 739** |
| Le/*le*^59,508^ | **59G, 202, 314, 508A, 1067** | *se*^385^/*se*^385^ | **357, 385TT, 428, 739** | Le/Le&*se*^385^/*se*^385^ | **59, 202, 314, 508, 1067/ 357, 385TT, 428, 739** |
| Le/*le*^59^ | **59G, 202, 314, 508, 1067** | Se/Se | **357, 385, 428, 739** | Le/Le&Se/Se | **59, 202, 314, 508, 1067/ 357, 385, 428, 739** |
| *le*^59,508^/*le*^59,508^ | **59GG, 202, 314, 508AA, 1067** | Se/*se*^357,385^ | **357C, 385T, 428,739** | Le/*le*^59,508^&Se/*se*^385^ | **59G, 202, 314, 508A, 1067/ 357, 385T, 428, 739** |
| Le/*le*^59,1067^ | **59G, 202, 314, 508, 1067A** | Se/Se^357^ | **357C, 385, 428, 739** | Le/Le&Se/*se*^357,385^ | **59, 202, 314, 508, 1067/ 357C, 385T, 428, 739** |
| Le/*le*^202,314^ | **59, 202C, 314T, 508, 1067** | Se^357^/Se^357^ | **357CC, 385, 428, 739** | Le/Le&Se/Se^357^ | **59, 202, 314, 508, 1067/ 357C, 385, 428, 739** |
| *le*^59,1067^/*le*^59,508^ | **59GG, 202, 314, 508A, 1067A** | *se*^385^/*se*^357,428,739^ | **357C, 385T, 428A, 739A** | Le/*le*^59^&Se/*se*^385^ | **59G, 202, 314, 508, 1067/ 357, 385T, 428, 739** |
| *le*^59^/*le*^202,314^ | **59G, 202C, 314T, 508, 1067** | Se^357^/*se*^357,385^ | **357CC, 385T, 428, 739** | Le/*le*^59,508^&Se/Se | **59G, 202, 314, 508A, 1067/ 357, 385, 428, 739** |
| *le*^59,1067^/*le*^59,1067^ | **59GG, 202, 314, 508, 1067AA** | Se^357^/*se*^357,428,739^ | **357CC, 385, 428A, 739A** | Le/*le*^59,508^&*se*^385^/*se*^385^ | **59G, 202, 314, 508A, 1067/ 357, 385TT, 428, 739** |
| *le*^59,508^/*le*^202,314*^ | **59G, 202C, 314T, 508A, 1067** | Se/*se*^357,428,739^ | **357C, 385, 428A, 739A** | *le*^59,508^/*le*^59,508^&*se*^385^/*se*^385^ | **59GG, 202, 314, 508AA, 1067/ 357, 385TT, 428, 739** |
| Le/*le*^1067^ | **59, 202, 314, 508, 1067A** |  |  | *le*^59,508^/*le*^59,508^&Se/*se*^385^ | **59GG, 202, 314, 508AA, 1067/ 357, 385T, 428, 739** |
| *le*^59^/*le*^59^ | **59GG, 202, 314, 508, 1067** |  |  | Le/*le*^59,508^&Se/Se^357^ | **59G, 202, 314, 508A, 1067/ 357C, 385, 428, 739** |
| *le*^59,1067^/*le*^202,314*^ | **59G, 202C, 314T, 508, 1067A** |  |  | Le/*le*^59,508^&Se/*se*^357,385^ | **59G, 202, 314, 508A, 1067/ 357C, 385T, 428, 739** |
| Le/*le*^508^ | **59, 202, 314, 508A, 1067** |  |  | Le/*le*^59,508^&*se*^385^/*se*^385^ | **59G, 202, 314, 508, 1067A/ 357, 385TT, 428, 739** |
|  |  |  |  | Le/*le*^59^&Se/Se | **59G, 202, 314, 508, 1067/ 357, 385, 428, 739** |
|  |  |  |  | Le/*le*^59,1067^&Se/*se*^385^ | **59G, 202, 314, 508, 1067A/ 357, 385T, 428, 739** |
|  |  |  |  | Le/*le*^202,314^&Se/Se | **59, 202C, 314T, 508, 1067/ 357, 385, 428, 739** |
|  |  |  |  | Le/Le&Se^357^/Se^357^ | **59, 202, 314, 508, 1067/ 357CC, 385, 428, 739** |
| * The characters followed by digits is used to represent a positional mononucleotide variation. Single character, heterozygous. Double characters, homozygous. | | | | | |

Supplementary Table 3. Comparison of Lewis and Secretor allele frequencies among different populations

| **Lewis gene alleles** | **Chines***  **(316)** | **Chinese A^a^**  **(GZ)**  **(154)** | **Chinese B^a^**  **(SY)**  **(138)** | **Taiwanese^b^ (137)** | **Korean^c^**  **(242)** | **Japanese^d^**  **(400)** | **Mongolian^e^ (50)** | **Amazon^f^**  **(150)** | **Xhosa^g^**  **(100)** | **Ghanaian^e^ (106)** | **Caucasian**  **A^e^**  **(100)** | **Caucasian**  **B^g^**  **(100)** |
| --- | --- | --- | --- | --- | --- | --- | --- | --- | --- | --- | --- | --- |
| Le | 72.94% | 67.50% | 75.00% | 68.20% | 73.10% | 68.90% | 56.00% | 49.70% | 50.00% | 42.00% | 70.50% | 67.50% |
| *le*^59,508^ | 14.72% | 14.00% | 14.50% | - | 22.30% | 24.80% | 24.00% | 15.30% | 31.00% | 18.90% | 1.50% | 1.00% |
| *le*^59^ | 4.75% | 2.60% | 1.10% | - | 1.00% | 0.50% | 0.00% | 29.00% | 0.00% | 0.00% | 0.00% | 2.00% |
| *le*^59,1067^ | 4.27% | 12.30% | 5.40% | - | 3.50% | 5.80% | 3.00% | 2.00% | 2.50% | 1.40% | 4.00% | 13.00% |
| *le*^202,314^ | 2.85% | 2.60% | 3.60% | 5.10% | - | - | 0.00% | - | 8.00% | 6.60% | 17.00% | 14.00% |
| *le*^1067^ | 0.32% | 1.00% | 0.40% | 13.90% | 0.00% | - | 0.00% | 2.00% | 0.00% | 0.00% | 0.00% | 0.50% |
| *le*^508^ | 0.16% | - | - | 12.80% | 0.00% | - | 0.00% | 0.70% | 0.00% | 0.00% | 0.00% | 0.00% |
| *le*^59,508,1067^ | 0.00% | - | - | - | - | - | 0.00% | 1.30% | 0.00% | 0.00% | 0.00% | 0.00% |
| **Secretor gene alleles** | **Chines* (316)** | **Chinese A^h^ (GZ)**  **(154)** | **Chinese B^h^ (SY)**  **(138)** | **Chinese C^i^ (HK)**  **(79)** | **Chinese D^j^ (Tibetan)**  **(80)** | **Taiwanese^k^ (124)** | **Thai (Taiwan)^k^ (70)** | **Japanese^d^**  **(400)** | **Japanese (Fukuoka)^m^ (141)** | **Korean A^l^**  **(348)** | **Korean**  **B^h^**  **(158)** | **Xhosa (South Africa)^n^ (100)** |
| *Se* | 46.20% | 13.98% | 15.95% | 5.10% | 3.80% | 53.63% | 41.40% | 13.90% | 10.30% | 20.80% | 10.13% | 9.90% |
| *se*^385^ | 40.19% | 0.00% | 0.00% | - | 0.00% | 44.35% | 53.60% | 0.00% | 0.00% | 7.20% | 0.00% | - |
| *Se*^357^ | 7.12% | 35.06% | 39.10% | 35.40% | 26.30% | - | - | 40.80% | 40.10% | 18.70% | 40.51% | 13.90% |
| *se*^357,385^ | 5.85% | 48.05% | 43.10% | 45.60% | 63.80% | - | - | 40.60% | 43.60% | 42.40% | 48.73% | 0.00% |
| *se*^357,428,739^ | 0.63% | - | - | - | - | - | - | 0.00% | - | - | - | - |
| *se*^428^ | 0.00% | 0.00% | 0.70% | 1.25% | 2.50% | 0.00% | 1.40% | 0.00% | 0.00% | - | 0.00% | 41.60% |
| *se*^fus^ | 0.00% | 0.00% | 0.00% | - | 0.00% | - | 0.00% | 4.70% | 5.70% | 10.80% | 0.63% | 0.00% |

*Population in present study. Symbol “-” represents not mentioned.

a: Liu et al. 1999 (10), b: Liu et al. 2000 (19), c: Park et al. 2010 (28), d: Narimatsu et al. 1998 (25), e: Soejima et al. 2009 (30), f: Corvelo et al. 2013 (16), g: Pang et al. 1998 (15), h: Liu et al. 1999 (23), i: Yip et al. 2007 (22), j: Pang et al. 2001 (29), k: Chang et al. 1999 (26), l: Park et al. 2005 (9), m: Koda et al. 1996 (6), n: Liu et al. 1998 (27).
